# Clustering protein functional families at large scale with hierarchical approaches

**DOI:** 10.1002/pro.5140

**Published:** 2024-08-15

**Authors:** Nicola Bordin, Harry Scholes, Clemens Rauer, Joel Roca‐Martínez, Ian Sillitoe, Christine Orengo

**Affiliations:** ^1^ Institute of Structural and Molecular Biology University College London London UK; ^2^ Universidad Autonoma de Madrid, Ciudad Universitaria de Cantoblanco Madrid Spain

**Keywords:** CATH, domain classification, embeddings, functional classification, FunFams

## Abstract

Proteins, fundamental to cellular activities, reveal their function and evolution through their structure and sequence. CATH functional families (FunFams) are coherent clusters of protein domain sequences in which the function is conserved across their members. The increasing volume and complexity of protein data enabled by large‐scale repositories like MGnify or AlphaFold Database requires more powerful approaches that can scale to the size of these new resources. In this work, we introduce MARC and FRAN, two algorithms developed to build upon and address limitations of GeMMA/FunFHMMER, our original methods developed to classify proteins with related functions using a hierarchical approach. We also present CATH‐eMMA, which uses embeddings or Foldseek distances to form relationship trees from distance matrices, reducing computational demands and handling various data types effectively. CATH‐eMMA offers a highly robust and much faster tool for clustering protein functions on a large scale, providing a new tool for future studies in protein function and evolution.

## 1 INTRODUCTION

Evolutionary changes in sequence and structure over millions of years led to the emergence of a variety of functions in proteins. These changes are often conserved if their impact does not affect survivability and can lead to an improvement in cellular fitness by increasing the performance of existing proteins, broadening the specificity, or by creating novel functionalities. Originating from a common evolutionary ancestor, the emergence of different functions is caused by a variety of events, both genetic and environmental. Genetic changes may include whole genome duplications, transposable element insertions, splicing effects, and single nucleotide polymorphisms, among others, while environmental perturbations may effect changes in germinal lines that are transferred to the next generations.

The location of these mutations and their selection is evident from the variability encountered in protein families, where the core of a globular protein domain is mostly conserved across time and the Tree of Life, with further embellishments and variability in noncore regions, active sites, or allosteric sites (Bordin et al., 2021; Chothia, 1992). From a sequence standpoint, these mutations are allowed only in regions that are not fundamentally affecting the folding stability of the protein. Using a combination of sequence and structural data with experimental and in silico approaches, various resources have classified proteins into homologous superfamilies containing members related by evolution, including CATH, SCOP2, SCOPe, and ECOD (Andreeva et al., 2014; Cheng et al., 2014; Fox et al., 2014; Sillitoe et al., 2021). The CATH protein structure classification database classifies in its 4.3 release over half a million protein domain structures in 6631 superfamilies, where each superfamily contains domains that are descendants of a common evolutionary ancestor. The largest 200 superfamilies in CATH, comprising over 62% of the domains in the database, are very diverse in function, having an average of 27 unique EC4 terms, 690 unique GO terms, as well as 11 structural clusters (in which relatives superimpose within 5 Å) per superfamily.

Several approaches have been developed over the years to capture and classify relatives having different functions across superfamilies of proteins. These can be based on multiple sequence alignments (MSA), network analyses, pairwise sequence analyses, coevolving residues in MSAs, and relationships between Hidden Markov Models (HMM). Early strategies inferred a phylogenetic tree from a multiple sequence alignment for each superfamily and subsequently split the tree to generate functional subgroups of proteins (Del Sol et al., 2003; Lichtarge et al., 1996; Sahraeian et al., 2015). Pairwise sequence alignments are used to generate networks of functional relationships, and analyses of signals from coevolutionary residues coupled with statistical analyses are used by numerous approaches (Mihaljević & Urban, 2020; Narayanan et al., 2017; Neuwald et al., 2018; Rivoire et al., 2016; Salinas & Ranganathan, 2018). For a comprehensive review on methods for functional classification of proteins refer, to Rauer et al. (2021).

In order to capture and exploit this wealth of functional diversity, an additional level in CATH was created: Functional Families, a subgrouping of homologous proteins in which relatives are likely to perform the same function (Das, Lee, et al., 2015). Functional Families, or FunFams in short, are generated by the GeMMA algorithm (genome modeling and model annotation). This first clusters sequences assigned to a given CATH superfamily by HMM hits, into clusters of relatives having at least 90% sequence identity and discards clusters that do not have at least one protein with an experimentally‐derived GO term in UniProt. These “starting clusters” are aligned and converted to Hidden Markov Models that are compared in an all‐versus‐all fashion using HH‐align. Clusters are iteratively merged, resulting in a tree of relationships between HMMs (Lee et al., 2010). The tree is subsequently repopulated by its original multiple alignments, traversed, and pruned in order to obtain the largest coherent subsets possible in which a putative function is conserved. The algorithm that performs this last step, FunFHMMER, looks for the presence of differentially conserved sets of residues between MSAs, likely to be related to specific functions differing between the alignment subsets (Das, Sillitoe, et al., 2015).

**FIGURE 1:**
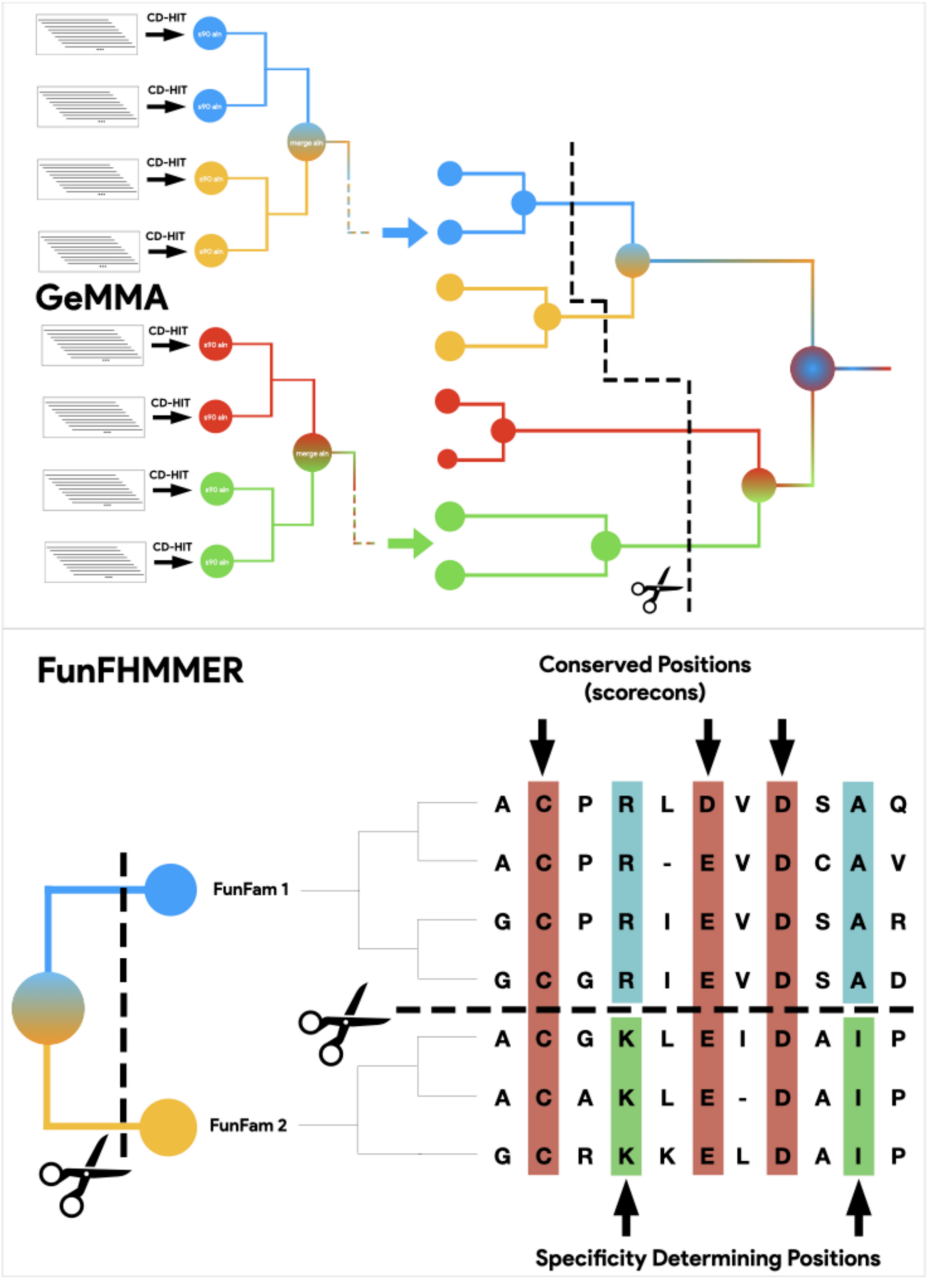
Genome modeling and model annotation (GeMMA) and FunFHMMER algorithms.

FunFHMMer determines the optimal partitioning of the GeMMA tree into subsets, each of which is a FunFam by operating on the MSAs of leaves and internal nodes (Figure 1).

This original approach led to the partitioning of 2620 CATH SuperFamilies (42%) in CATH version 4.2 into 67,598 FunFams comprising 8.6 million domain sequences (Sillitoe et al., 2021).

During the process of generating Functional Families for CATH version 4.2, we encountered the upper bound of the original GeMMA/FunFHMMer approach at 5000 starting clusters, and with an ever‐growing number of sequences in UniProt being mapped to CATH superfamilies, we explored various avenues to improve the outcome of functional clustering for protein domains. This manuscript explores novel approaches in protein functional clustering, including data prepartitioning from CATH v4.3, algorithm optimization, and a search for a more modern and scalable approach beyond Hidden Markov Models.

## 2 RESULTS

### 2.1 CATH‐MARC (multi‐domain architecture clustering)

**FIGURE 2:**
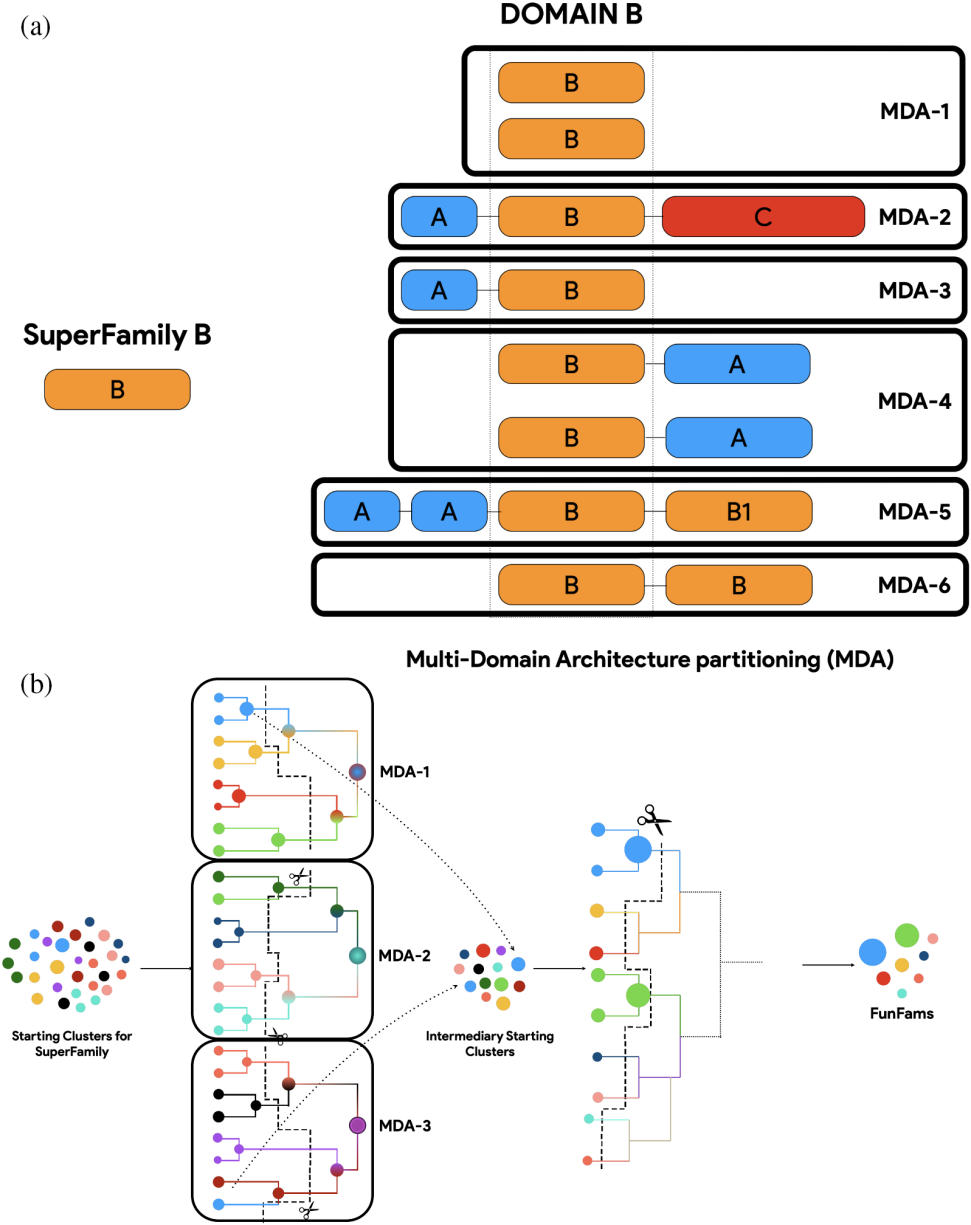
Multi‐domain architecture clustering (CATH‐MARC) algorithm. (a) Multi‐domain architecture partitioning of CATH SuperFamily. (b) Multiple iterations of genome modeling and model annotation (GeMMA)‐FunFHMMer with FunFams as intermediate starting clusters.

The composition and order of domains in a protein is often more conserved than their structures (Apic et al., 2001; Björklund et al., 2005). For example, particular arrangements such as a beta propellor followed by a spaH domain can be found in eukaryotic nuclear pore complexes and proto‐membranes in the Planctomycetes‐Verrucomicrobiae‐Chlamydia, with as little as 4% sequence identity conserved between these SuperKingdoms (Devos et al., 2004; Santarella‐Mellwig et al., 2010). These arrangements can be described as the Multi‐Domain Architecture (MDA) of a protein (Figure 2a). CATH‐MARC–multi domain architecture clustering, an improved version of the GeMMA/FunFHMMEr protocol used to generate the v4.3 release of CATH‐FunFams, is based on the principle that proteins having different MDAs are likely to have rather different functions; therefore, data can be prepartitioned more effectively by segregating them ab‐initio (Bashton & Chothia, 2007; Dessailly et al., 2009; Lees et al., 2014; Yu et al., 2019). CATH domain assignments on UniProt sequences is available via the ancillary Gene3D resource (Lewis et al., 2018), and their order in the protein sequence are provided as “MDA strings” (i.e., a protein with a P‐loop domain at the N‐ter, followed by an immunoglobulin, and a “High‐signature, UspA, PP‐ATPase” (HUP) domain at the C‐ter will have the MDA string 3.40.50.300–2.40.60.10–3.40.50.620). The Gene3D MDA assignments are therefore first grouped by MDA, provided there are at least a million sequences, before the initial clustering and GO‐based selection. For smaller sets of sequences sharing the same MDA, these are collected in a single set. The algorithm differs from the original implementation of GeMMA/FunFHMMer as the FunFams generated from each MDA are then pooled and used as starting clusters for a final iteration of the algorithm (Figure 2b).

This approach, when applied to the release v4.3 of CATH, led to an overall expansion in the number of FunFams from 68,065 in v4.2 to 212,872, covering an additional 1645 superfamilies (a 61% increase in coverage by superfamily and a 300% increase in total sequences classified). However, this increase in coverage did not hinder EC purity since the overall FunFam purity level and diversity of positions scores (DOPS) increased between the previous release and version 4.3 (Sillitoe et al., 2021; Valdar, 2002). Another key advantage of this approach is its parallelization, as each MDA can be processed separately and reduces the time and memory footprint, reducing the processing time from 6 months (CATH v4.2) to 6 weeks (CATH v4.3) despite a significant increase in sequences classified in CATH FunFams.

### 2.2 Functional families generation by RANdom splitting (FRAN)

**FIGURE 3:**
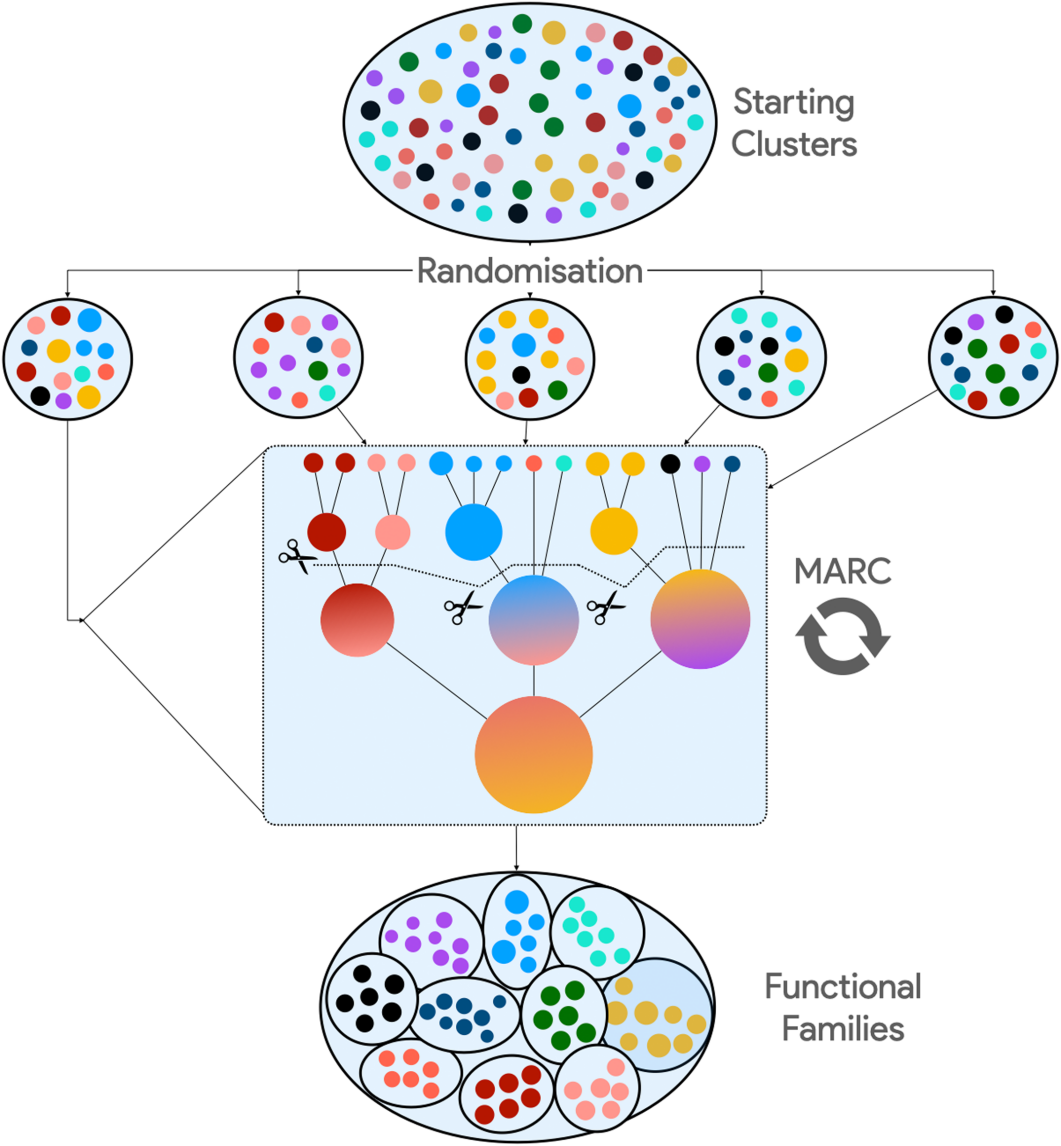
Functional families by random splitting of starting clusters.

The prepartitioning of domain sequence data introduced with CATH‐MARC improves functional coherence and allows each multi‐domain architecture to be treated as an individual project for the first iteration of tree building, reducing the computational footprint by a factor of 4 (from 6 months to 6 weeks). Although a significant improvement, a dozen superfamilies, including the P loops and immunoglobulins, have individual MDA sets containing over 2 million sequences and tens of thousands of starting clusters, and these numbers will increase further when metagenomic sequences are classified into superfamilies for functional studies. The Alpha/Beta Hydrolase superfamily, containing various enzymes with environmental and industrial applications, is represented in CATH with 3809 structures and over 800,000 domain sequences. These enzymes present an extreme variety in their structure and substrates, with close to 2000 unique Functional Families in CATH v4.3. By scanning MGnify sequences (Mitchell et al., 2019) against the HMMs built from each S95 representative, the initial number of starting clusters for the Alpha/Beta Hydrolase is close to 1.5 million, three orders of magnitude larger than the current limitation of MARC. A successful strategy for clustering functional groups in these large metagenomic datasets relies on randomly partitioning the initial sequence set, building trees from these randomly assigned sets, and relying on a second iteration of tree building and cutting to merge outliers correctly. Functional Families generation by RANdom splitting (FRAN) generates starting clusters at 90% sequence identity, generates projects, and fills each with a predetermined number of clusters selected randomly from the pool of S90. Each project is then processed using the MARC protocol, with the Functional Families resulting from the first iteration being used as the starting clusters of the second iteration of the tree‐building and cutting algorithm (Figure 3).

The randomization involved in the first round of the algorithm affects slightly the ultimate purity of the resulting Functional Families, as the second iteration of the algorithm processes most outliers in each starting cluster introduced by the randomization process.

### 2.3 CATH‐eMMA

**FIGURE 4:**
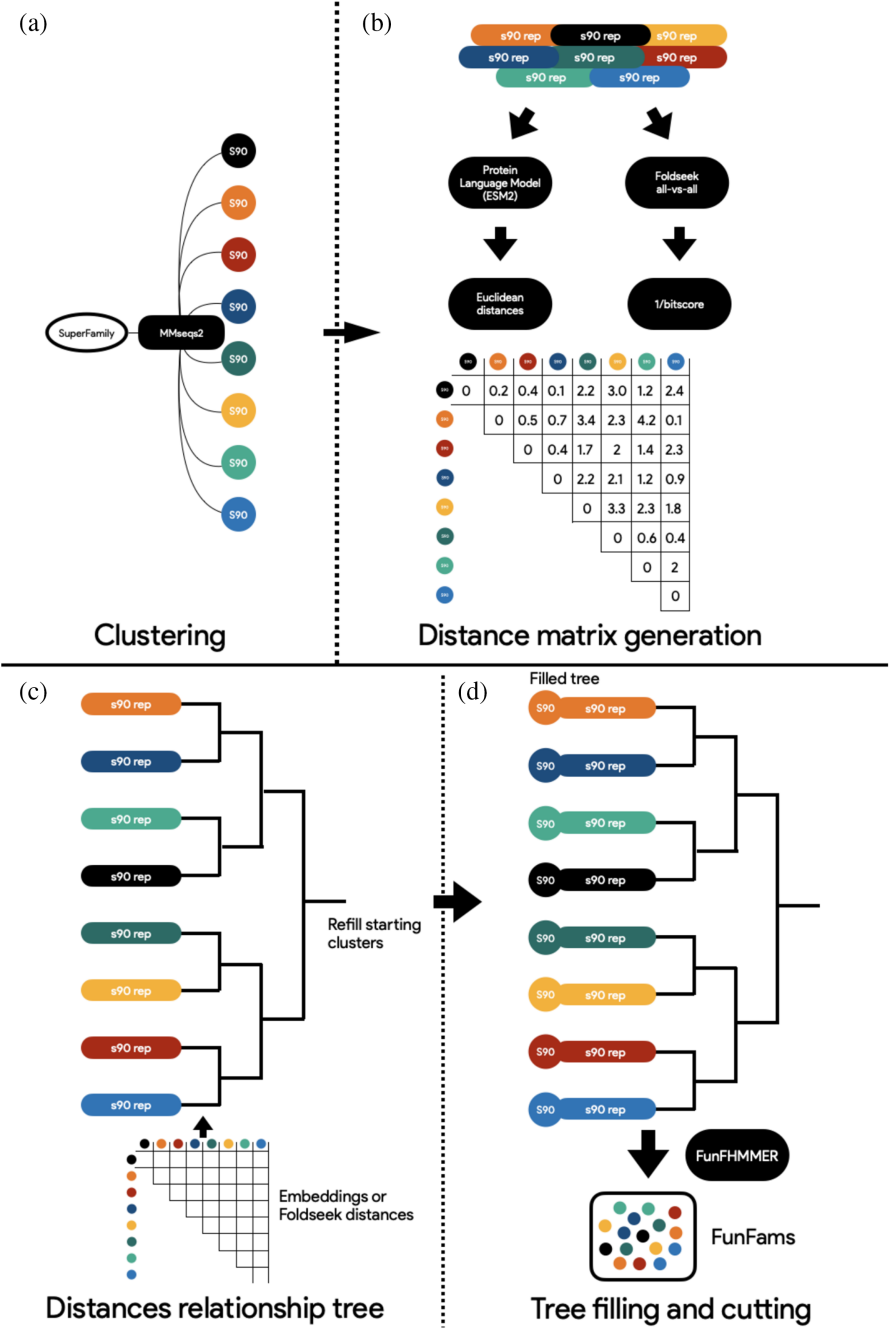
CATH‐eMMA algorithm for classifying functions using embeddings or Foldseek distances.

Recent advances in protein language models (Elnaggar et al., 2020; Heinzinger et al., 2022; Heinzinger et al., 2023; Rives et al., 2021; Yu et al., 2023) and ultra‐fast protein aligners based on the encoding of protein structure conformations (van Kempen et al., 2023) enabled the use of embeddings and structural alphabets as features for determining evolutionary distances as well as sequence and structural similarities. Proximity in hyperdimensional space between the embeddings of two proteins can be used to infer common characteristics, including structure, function, and evolutionary history (Bordin et al., 2023; Durairaj et al., 2023; Gligorijević et al., 2021; Kilinc et al., 2023; Nallapareddy et al., 2023). As GeMMA relies on a very large similarity matrix based on Hidden Markov Model distances, we examined whether it would be effective to replace the core engine of the algorithm with a source‐agnostic half‐matrix of distances based on embedding (e.g., cosine, Euclidean, and Manhattan) or structural distances (1/bitscore, RMSD), as well as modernizing the pipeline environment from Perl to Python. The resulting algorithm, eMMA (a portmanteau of embeddings and GeMMA), is a Python based CLI pipeline that interfaces with local and distributed platforms (i.e., SGE) (https://github.com/UCLOrengoGroup/eMMA). CATH‐eMMA runs modified versions of GeMMA and FunFHMMER, with various advantages over its predecessors (MARC, FRAN, and the original implementation of GeMMA/FunFHMMER). The first step in the eMMA algorithm substitutes CD‐HIT (Li & Godzik, 2006) with MMseqs (Steinegger & Söding, 2017), a more modern, faster, and scalable aligner while retaining the same clustering behavior as the former (Figure 4a: Clustering). A significant reduction in memory consumption is based on the second step, which involves generating embeddings or structure‐based distance half‐matrices only for the cluster representatives, removing the need to store the entire alignment in memory (Figure 4b: Distance matrix generation). A modified version of GeMMA then stores the matrix in memory, removing the need to align, convert, and search HMMs, thus reducing the time needed to create the relationship tree (Figure 4c: Distances relationship tree). Ultimately, each starting cluster is refilled from its representative, and a version of FunFHMMer modified to process distances instead of E‐values traverses the GeMMA tree to generate Functional Families (Figure 4d: Tree filling and cutting). The advantages of the eMMA approach are various, as it requires less memory, less computing time, and can be fed diverse sources of data.

### 2.4 Benchmarks

To benchmark the efficacy and behavior of each approach (GeMMA/FunFHMMER, MARC, FRAN, and eMMA), we clustered the HUPs superfamily in CATH (3.40.50.620). We selected this superfamily as it is very diverse from a structural and functional standpoint, and most of its sequences have extensive functional annotations on UniProt. The HUP domain superfamily has 1685 structures in CATH and over 650 thousand domain relatives in UniProt, as predicted by Gene3D, and exhibits extreme diversity with over 21 structural clusters (relatives superpose within 9 Å), 55 different EC terms, over 600 GO terms, and relatives found in 62 MDA contexts in over 26 thousand organisms. After clustering relatives at 90% sequence identity, we proceeded only with clusters containing experimentally derived EC annotations. Out of 650 thousand HUP sequences, only 40,226 have experimental EC characterization.

**FIGURE 5:**
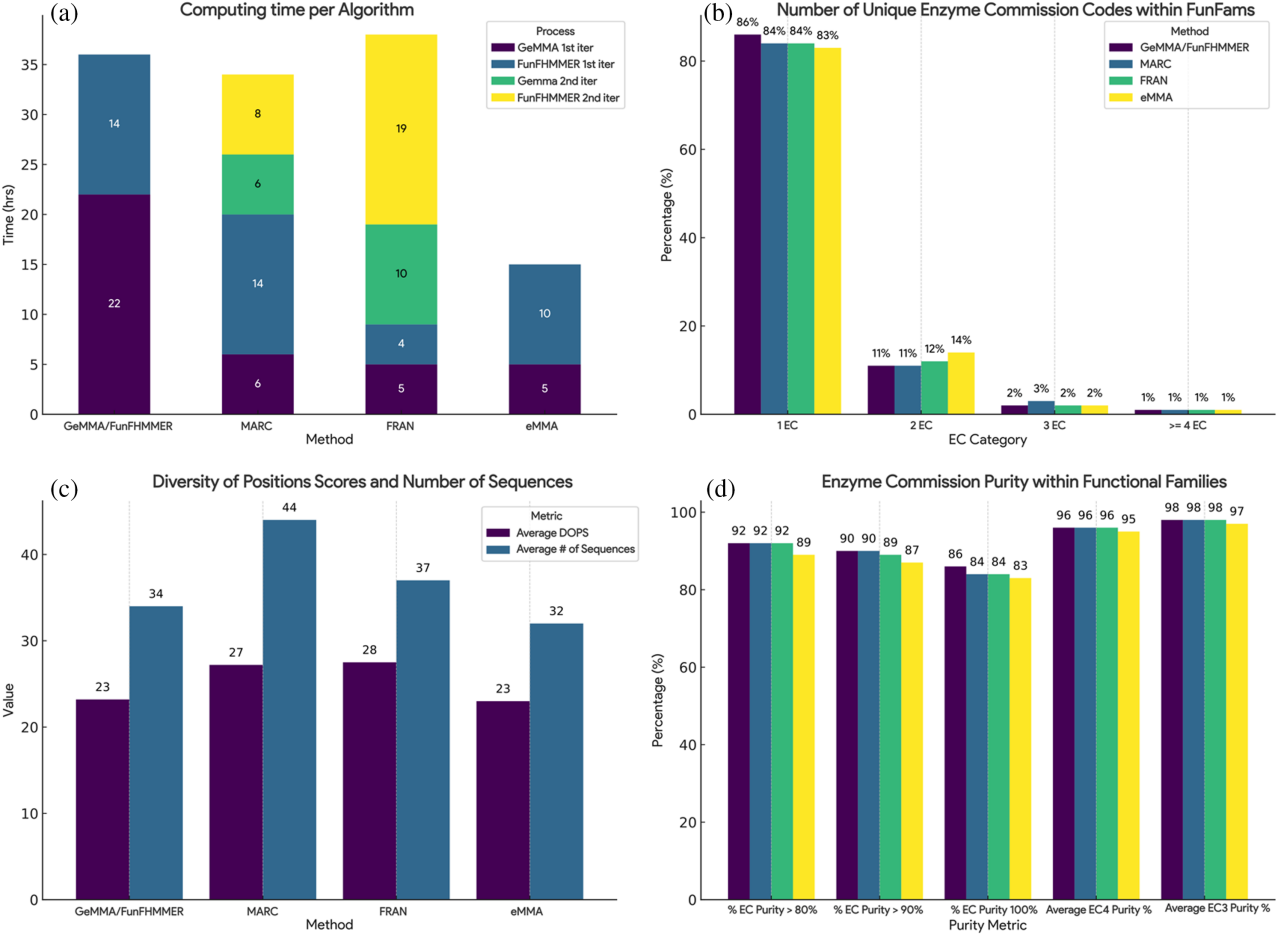
(a) Computing time for each step of the algorithms. (b) Number of unique enzyme commission codes within functional families. Proportion over total. (c) Average diversity of positions scores (DOPS) score and number of sequences within each functional family. (d) Enzyme commission code purity. Proportion over total of FunFams with an EC purity over 80%, 90%, and 100%, as well as the average EC4 and EC3 purity.

MARC and FRAN starting cluster data were prepartitioned according to MDA or randomly split into an equivalent number of partitions, while starting clusters for GeMMA/FunFHMMER and eMMA were processed in bulk and classified into functional families (Table 1). Using the algorithms as described above, we generated FunFams and benchmarked their quality using the EC purity and diversity of positions (DOPs) Score (Valdar, 2002) (Figure 5). The DOPs values provide information on the sequence diversity of the clusters. These are important metrics to consider as FunFam multiple sequence alignments (MSAs) are used to identify conserved residues likely to be linked to functional sites. Clustering too narrowly may increase purity, but at the expense of losing information on conserved sites, as conservation can only be measured in diverse (information rich) MSAs.

**TABLE 1:** Starting clusters and resulting FunFams for each algorithm applied to the HUPs SuperFamily.

Algorithm	Starting clusters	FunFams 1st iteration	FunFams 2nd iteration
GeMMA/FunFHMMER	3693	1200	1200
MARC	3723	1221	921
FRAN	3693	2789	1099
eMMA	3697	1262	1262

Abbreviations: FRAN, functional families generation by RANdom splitting; FunFams, functional families; GeMMA, Genome modeling and model annotation; MARC, multi‐domain architecture clustering.

All methods have comparable performance and purity with the original implementation of the GeMMA/FunFHMMER algorithm while improving in certain areas. By exploiting Multi‐Domain Architecture information, MARC improved slightly on the EC purity of GeMMA/FunFHMMEr (Figure 5b,d), while drastically reducing the time and computing requirements of the first iteration of GeMMA (Figure 5a). The first iteration of FunFHMMEr is consistent across these two methods, with a comparable computing time to traverse and cut each tree. The second iteration of GeMMA and FunFHMMEr in MARC takes significantly less time, as Functional Families sequences are already in close vicinity and the pooling process reduces significantly the search space in comparison with the single iteration of GeMMA/FunFHMMER in the original algorithm.

FRAN has a very fast first iteration of GeMMA and FunFHMMER due to the size constraint on their starting cluster numbers, although the resulting FunFams are almost twice the number of the other methods, suggesting that the random splitting is creating a very diverse tree, which is particularly difficult for FunFHMMER to traverse and merge individual functions (Figure 5a). The second iteration of FunFHMMER is the longest across all algorithms, due to the effort involved in comparing and aligning very diverse sets of sequences.

FunFams generated by eMMA have a slight degradation in EC purity due to the algorithm relying on the signal from comparing single sequence representatives for the clusters rather than HMM‐HMM comparisons of the multiple sequence alignments from the clusters. Ultimately the tradeoff between memory/speed and purity is favorable, as potentially these EC impurities could be addressed by additional iterations of the algorithm, and future work will investigate additional strategies for comparing the clusters to improve purity.

The diversity of positions score retrieved from scorecons (Valdar, 2002) shows comparable alignment diversity for FunFams generated by GeMMA/FunFHMMER and eMMA, and for FunFams generated by MARC and FRAN (Figure 5c). FunFam eMMA shows slightly lower values than MARC and FRAN, suggesting that more work is needed to optimize merging of the clusters in order to improve diversity and information content whilst ensuring high levels of purity are maintained.

## 3 DISCUSSION

The classification of protein sequences into sets having coherent functions is valuable for a variety of downstream applications, including the detection of variants, the identification of residues that are differentially conserved across functional families, and the assignment of function to uncharacterized proteins. Functional families have been used successfully in the past to assign putative functions to targets in various rounds of the CAFA functional annotation evaluation (Jiang et al., 2016; Radivojac et al., 2013; Zhou et al., 2019), have identified putative variants in cancer (Sillitoe et al., 2021), and classified Kinases according to their specificity‐determining positions (Adeyelu et al., 2023). They are often used as a proxy for assigning functions to whole proteins while being domain‐based annotations only. The conserved residues across a FunFam reflect the domain's environment in a multi‐domain architecture, so a FunFam assignment can often be used to extrapolate a higher‐level function to the whole protein (Das, Lee, et al., 2015; Rentzsch & Orengo, 2013). Functional impurities in FunFams can be ascribed to either the presence of uncertain EC4 annotations (e.g., EC:3.4.12.‐) in proteins within each alignment or the incorrect merging of two subsets of sequences with different functions with FunFMMER.

With the emergence of extremely large datasets from metagenomes (Mitchell et al., 2019), and the availability of structures for the majority of proteins in UniProt via the AlphaFold database (Varadi et al., 2022), this treasure trove of evolutionary information is both a boon and a bane for these algorithms, as larger sequence sets contain many unexplored portions of functional space but are increasingly becoming intractable by methods that could cope with hundreds of thousands or a couple of millions of sequences at best. Recent works (Barrio‐Hernandez et al., 2023; Durairaj et al., 2023) have exploited novel structural clustering capabilities by Foldseek (van Kempen et al., 2023) to reduce the search space for the entirety of the structure space in UniProt, but the number of cluster representatives is still in tens of millions, and these clusters are based on chain‐level groupings, with domain‐level annotations still missing from the general picture. Annotations in “dark” regions of the protein structure space are very sparse, while representing large swaths of it, particularly in bacterial genomes (Perdigão et al., 2015). Furthermore, embedding‐based annotation tools are rapidly overtaking HMMs as the state of the art for functional inference (Kilinc et al., 2023), and BLAST‐like embedding‐based tools will enable a rapid and accurate way to search these large regions of the “unknownome.” (Hamamsy et al., 2023) The main pitfall of embedding‐based methods is the lack of embedding databases; therefore, these new search tools have a limited search space. Future improvements are likely to involve exploitation of both homology and embedding‐based information in a more efficient manner to cluster the protein function space using the structure space as a scaffold to focus our functional annotation efforts.

## 4 METHODS

### 4.1 Sequence data

Protein sequences for the HUPs Superfamily in CATH v4.3 (http://www.cathdb.info/version/latest/superfamily/3.40.50.620) were retrieved from the latest release of Gene3D (v21) and their GO and EC annotations downloaded from UniProt using the Retrieve/ID Mapping service (https://www.uniprot.org/id-mapping).

### 4.2 Clustering

Protein domain sequences were clustered at 90% sequence identity using CD‐HIT (Li & Godzik, 2006) for GeMMA/FunFHMMER, MARC, and FRAN. Equivalent behavior was achieved using MMseqs2 (Steinegger & Söding, 2017) by following the documentation to replicate CD‐HIT clustering in MMseqs2 at https://github.com/soedinglab/mmseqs2/wiki#how‐do‐parameters‐of‐cd‐hit‐relate‐to‐mmseqs2.

### 4.3 Tree generation

Trees for GeMMA/FunFHMMER, MARC, and FRAN were generated using GeMMA (https://github.com/UCL/cath-gemma) (Lee et al., 2010). A modified version of GeMMA used to generate trees from source agnostic matrices is part of the new eMMA Github repository (https://github.com/UCLOrengoGroup/eMMA).

### 4.4 Tree traversing and cutting

Trees for GeMMA/FunFHMMER, MARC, and FRAN were traversed and cut into individual Functional Families using FunFHMMER version 2.2 (https://github.com/UCL/cath-funfhmmer/tree/funfhmmer_v2.2) (Das, Lee, et al., 2015). A version of FunFHMMER modified to process non‐E‐value‐based distance trees is available at https://github.com/UCLOrengoGroup/eMMA-FunFHMMER.

### 4.5 Embeddings from protein language models

Embeddings were generated using the dedicated CLI module in eMMA (https://github.com/UCLOrengoGroup/eMMA), which relies on the embedding generation capability of the ESM2 protein language model (esm2_t33_650M_UR50D) developed by MetaAI and available at https://github.com/facebookresearch/esm (Rives et al., 2021).

### 4.6 Alignment evaluation

Alignment quality metrics such as diversity of positions scores (DOPs) were calculated for each set of FunFams. DOPs rank alignment diversity by measuring the proportion of conserved positions via scorecons (conservation scores) and return a value from 0 (no variability, uninformative alignment) to 100 (highly diverse, informative alignment). DOPs is calculated as part of the implementation of scorecons (Valdar, 2002) included in the cathpy python package available at https://cathpy.readthedocs.io/en/latest/.

## AUTHOR CONTRIBUTIONS

**Nicola Bordin:** Conceptualization; investigation; writing – original draft; methodology; validation; visualization; writing – review and editing; software. **Harry Scholes:** Methodology; software; conceptualization; investigation; validation. **Clemens Rauer:** Conceptualization; investigation; methodology; validation; software. **Joel Roca‐Martinez:** software, validation. **Ian Sillitoe:** Methodology; software. **Christine Orengo:** Conceptualization; investigation; funding acquisition; writing – review and editing; project administration; supervision; resources.

## Data Availability

The eMMA Python pipeline is freely available on Github at https://github.com/UCLOrengoGroup/eMMA. The eMMA version of FunFHMMER is available at https://github.com/UCLOrengoGroup/eMMA-FunFHMMER. Benchmark code and data, including the embeddings for the HUPs superfamily is available on Zenodo at https://zenodo.org/doi/10.5281/zenodo.8425747.
